# Needle Catheter Jejunostomy in Patients Undergoing Surgery for Upper Gastrointestinal and Pancreato-Biliary Cancer–Impact on Nutritional and Clinical Outcome in the Early and Late Postoperative Period

**DOI:** 10.3390/nu12092564

**Published:** 2020-08-25

**Authors:** Maria Wobith, Lena Wehle, Delia Haberzettl, Ali Acikgöz, Arved Weimann

**Affiliations:** Clinical Nutrition Unit of the Department of General, Visceral, and Oncological Surgery, Klinikum St. Georg gGmbH Leipzig, 04103 Leipzig, Germany; maria.wobith@sanktgeorg.de (M.W.); lena.wehle@web.de (L.W.); delia.haberzettl@sanktgeorg.de (D.H.); ali.acikgoez@sanktgeorg.de (A.A.)

**Keywords:** needle catheter jejunostomy, malnutrition, upper gastrointestinal cancer surgery, nutritional status, recovery

## Abstract

The metabolic risk for patients undergoing abdominal cancer resection increases in the perioperative period and malnutrition may be observed. In order to prevent further weight loss, the guidelines recommend for high-risk patients the placement of a needle catheter jejunostomy (NCJ) for supplementing enteral feeding in the early and late postoperative period. Our aim was to evaluate the safety of NCJ placement and its potential benefits regarding the nutritional status in the postoperative course. We retrospectively analyzed patients undergoing surgery for upper gastrointestinal cancer, such as esophageal, gastric, and pancreato-biliary cancer, and NCJ placement during the operation. The nutritional parameters body mass index (BMI), perioperative weight loss, phase angle measured by bioelectrical impedance analysis (BIA) and the clinical outcome were assessed perioperatively and during follow-up visits 1 to 3 months and 4 to 6 months after surgery. In 102 patients a NCJ was placed between January 2006 and December 2016. Follow-up visits 1 to 3 months and 4 to 6 months after surgery were performed in 90 patients and 88 patients, respectively. No severe complications were seen after the NCJ placement. The supplementing enteral nutrition via NCJ did not improve the nutritional status of the patients postoperatively. There was a significant postoperative decline of weight and phase angle, especially in the first to third month after surgery, which could be stabilized until 4–6 months after surgery. Placement of NCJ is safe. In patients with upper gastrointestinal and pancreato-biliary cancer, supplementing enteral nutrition during the postoperative course and continued after discharge may attenuate unavoidable weight loss and a reduction of body cell mass within the first six months.

## 1. Introduction

In the time of Enhanced Recovery after Surgery programs (ERAS), perioperative nutrition therapy seems to be very “traditional” and even redundant [1]. Early oral feeding is feasible even after esophagectomy and without impact on the incidence and severity of postoperative complications [2].

Nevertheless, abdominal cancer patients, with special regard to those with cancer in the upper gastrointestinal and hepatopancreatobiliary tract, bear a high metabolic risk for malnutrition. Between 32% and 87% of patients suffer from weight loss at the time of diagnosis [3]. The degree of malnutrition may be related to the type of cancer, stage and location [3]. Especially in patients with advanced cancer the prevalence of malnutrition may be as high, with 80–85% in pancreatic cancer, 65–85% in gastric cancer, and 60–80% in esophageal cancer [3], strongly associated with negative clinical outcome. Additionally, the metabolic risk increases with the age of patients [4]. Major abdominal surgery is the treatment of cure often as part of a multimodal therapy [5]. Due to functional changes after surgery and adjuvant radio-/chemotherapy, oral intake may be often limited for weeks and months and below the caloric needs. Long-lasting weight loss has to be awaited [6]. In a prospective study of malabsorption and malnutrition after esophageal and gastric surgery, a weight loss of more than 15% at six months was observed in 20% of the patients, while weight loss of more than 10% even occurred in all 45 disease-free patients [7]. Furthermore, impaired nutritional status by muscle loss, so called sarcopenia, has been also shown to be a predictor for long-term cancer survival [8].

Many studies have shown the feasibility, safety, and clinical benefits of needle catheter jejunostomy (NCJ) as long lasting access for enteral nutrition [9,10,11]. Life-threatening complications like bowel strangulation and ischemic necrosis have been also reported [12,13,14]. The guidelines of the European Society for Clinical Nutrition and Surgery (ESPEN) in surgery recommend: “with special regard to malnourished patients placement of a nasojejunal tube (NJ) or needle catheter jejunostomy (NCJ) should be considered for all candidates for tube feeding undergoing major upper GI- and pancreatic surgery (B) (BM)” [5].

Because nasojejunal and nasoduodenal tubes are associated with a significant rate of early accidental dislodgement [15,16], the ESPEN guideline group also considers for patients at nutritional risk, “feeding jejunostomy may be superior to nasojejunal or -duodenal tubes”. In these patients it might be reasonable to leave NCJ and to continue nutritional support therapy after discharge [5].

Despite these recommendations, placement of a NCJ during surgery in risk patients is not regularly done. An alternative to supplement nutrition is the parenteral route by a central venous catheter. Data is sparse regarding post-discharge enteral supplementation via NCJ and the early and late complications [17]. This retrospective analysis explores the impact of NCJ placement on postoperative complications, and the nutritional status of the patients undergoing surgery for upper gastrointestinal cancer is investigated.

## 2. Methods

We retrospectively analyzed 102 patients undergoing surgery for cancer of the upper gastrointestinal tract between January 2006 and December 2016, who had undergone NCJ placement during surgery and had been observed after discharge for four to six months after surgery. Study approval including the processing of personal data was obtained from the Ethical Review Board of the Saxonian Medical Association (EK-BR-63/20-1).

The nutritional status of the patients was assessed by the BMI and bioelectrical impedance analysis (BIA) preoperatively, 1 to 3 months postoperatively and 4 to 6 months postoperatively.

Body weight was measured in kg using a calibrated medical scale (SOEHNLE Typ 2790, Soehnle-Professional GmbH & Co. KG, 71536 Murrhardt, Germany) and height in m. Body mass index (BMI) was calculated weight (kg)/height (m^2^).

For BIA a bioelectrical impedance analyzer Nutriguard-M (Data Input GmbH, 60487 Frankfurt, Germany) was used with 800 µA current and 50 kHz signal. Measurement was performed according to the standard technique in the fasting patient after emptying the urine bladder in the morning. From tissue resistance (R) and reactance (Xc) phase angle (α) could be calculated using the company software α = arctan (Xc/R) × 180°/π.

The nutritional status was defined by the BMI, weight loss and the phase angle. A phase angle between 5.0° and 7.9° complies with a normal nutritional status, whereas a phase angle less than 5° is an indicator for an underlying disease, a malnutrition or physical immobility [18].

Every patient received immunomodulating nutrition with arginine, omega-3-fatty-acids and ribonucleotides for five to seven days before surgery to reduce infectious complications and the length of hospital stay. During surgery, Freka^®^ NCJ from Fresenius Kabi was placed according to standard technique [19]. Subsequently, 24 h after surgery the tube feeding started with an enteral standard nutrition with 1.0 kcal/mL. Using prokinetic medication the infusion rate was slowly increased from 10 mL/h to approximately 80 mL/h depending on the tolerance of the patient. At the fifth postoperative day the patients received 500 kcal/day on average. If the oral nutrition was highly limited, the daily calories were increased up to 750–1500 kcal/day. Food intake was not pushed and “traditionally” started with clear fluids followed by liquid diet like oral nutritional supplements. In case energy requirements were not covered via the oral and enteral route alone (< 50% for seven days), supplemental parenteral nutrition was administered in line with the ESPEN guidelines [5].

All the patients had nutritional counselling and training regarding the handling of NCJ before discharge. Then, supplementing enteral nutrition was continued after discharge using a standard diet (1 kcal/mL) with a daily amount of 500–750 mL.

The endpoints length of hospital stay, length of NCJ placement, NCJ-associated complication rate and postoperative complication rate in Clavien-Dindo were evaluated [20]. Major complications were defined as Clavien-Dindo ≥ IIIb.

Statistical analysis was carried out with SPSS for Microsoft Windows, version 21. Continuous variables were shown as median and average. Normal distribution of variables was evaluated by the Shapiro-Wilk tests. Because there was almost no normal distribution of the variables, we used only nonparametric tests to compare the samples. The significance of the nutritional parameters with repetitive measurements was tested by the Friedman-Test. A *p*-value of less than 0.05 was referred to as statistically significant.

## 3. Results

In 102 patients a NCJ was placed during the esophageal, gastric, or pancreatic resection. In 8 of these patients the histopathological finding showed no malignancy, all these patients were suspected to have pancreatic cancer. In Table 1 and Table 2 the cancer stages, patient characteristics, surgical procedures, and clinical outcome parameters are summarized. Gastric and esophageal carcinomas were the most common indications for NCJ placement. Median length of hospital stay was 22 days (11 to 72 days). Complications occurred in 44.1% of the patients, no mortality. Major complications according to Clavien-Dindo ≥ IIIb were seen in 10.8% of patients. In total, 39 complications limiting an oral nutrition were observed, as shown in Table 3. NCJ associated complications were seen in 14 of the patients (13.7%). The most common complications were early dislocation (*n* = 10, 9.8%) and occlusion (*n* = 4, 3.9%) of the NCJ. In two cases guide wire aided placement of a new tube was performed through the old channel in the abdominal wall.

A postoperative supplementing enteral nutrition of 500 kcal/day was administered to 89.2% of the patients. Meanwhile 9.8% of patients required 750 to 1.000 kcal/day by NCJ, because the oral calory intake was not sufficient. In average the NCJ stayed for 118 days in the patient (15 to 1150 days).

Out of 102 patients, 90 could be enterally fed after discharge and showed up at the follow-up visits 1 to 3 months after surgery and 88 of those patients 4 to 6 months postoperatively. Preoperatively, the mean BMI was 26.4 kg/m^2^ (19.2 to 40.0 kg/m^2^) and it decreased after 1 to 3 months after surgery. After 4 to 6 months, there was no further decrease of the BMI, and it remained at a mean of 24.2 kg/m^2^ (17.1 to 31.9 kg/m^2^). Despite the daily enteral supplementation of 500 to 1.500 kcal per day, 95.6% of the patients lost weight during the first to third month after surgery. In the second follow-up visit 4 to 6 months after surgery, the proportion of patients with weight loss decreased to 89.8%. The mean postoperative weight loss was 7.7% and did not decrease during the follow-up time. A severe weight loss (>10%) after 1 to 3 months postoperatively was seen in 27.8% of the patients. After 4 to 6 months there was an even higher proportion of patients with severe weight loss with 39.8%. A weight loss of more than 15% after 1 to 3 months was seen in 5.6% and after 4 to 6 months in 13.6%. Preoperatively, the mean phase angle was slightly decreased with 4.7°, after 1 to 3 months it further decreased to 4.1° and after 4 to 6 months to 4.2°, respectively. At 6 months only 17 patients (19.3%) had a phase angle > 5°, while in 34 patients (38.6%) it was 4 – 4.9°. In 37 patients (42.1%) the phase angle was considerably reduced with < 4° (*n* = 7, 7.8% < 3) indicating malnutrition. The nutritional parameters preoperatively and in the postoperative period are shown in Table 4.

## 4. Discussion

After major abdominal surgery, especially in the upper gastrointestinal tract such as esophageal, gastric, and pancreatic cancer resections, oral intake will be often limited for weeks and months. With special regard to esophageal surgery, long lasting continuous weight loss has to be awaited for up to three years [21]. Overweight patients are at special risk [22,23]. In a systematic review of 18 studies more than half of the patients lost more than 10% of body weight in 12 months [24]. This may be attributed to a bariatric effect of cancer surgery including ghrelin deficiency [25]. Furthermore, body composition as sarcopenia may be considered a predictor for long-term survival [8,26,27].

According to a recent Cochrane Review, evidence to support the use of ghrelin in this group of patients is still insufficient [28]. Therefore, the ESPEN guidelines recommend supplementing enteral nutrition for all candidates for tube feeding undergoing major upper GI- and pancreatic surgery, even in case of an uneventful postoperative course [5]. It is not surprising that at special risk are patients with a complicated course and considerable deterioration of the nutritional status [29].

Some authors consider routine use of NCJ an overtreatment and propose consideration of NCJ only in real high-risk patients [30,31,32]. Others recommend it as a routine procedure for every patient undergoing esophagectomy [33,34,35]. For the short-term use during hospitalization, nasojejunal tubes seem to be more suitable [36]. Therefore, the major benefits of NCJ may be awaited in malnourished patients and those with anastomotic leakage of the esophago-gastrostomy, esophago-jejunostomy, and pancreato-jejunostomy, bearing safe access for long-standing enteral nutrition including home care.

The present study showed that in a special group of nutritional risk patients after esophagectomy, gastrectomy and pancreatic resection that enteral nutrition during the postoperative course and continued after discharge may attenuate unavoidable weight loss and reduction of body cell mass within the first six months. Furthermore, NCJ was safe even in use for several months after discharge.

Severe complications (Clavien-Dindo ≥ IIIb) were observed in 10.8% of the patients. Ligthart-Melis et al. observed Clavien-Dindo ≥ IIIb complications in patients undergoing esophageal resection after neoadjuvant treatment in 32% (*n* = 37) of patients with intensive nutritional therapy including NCJ, whereas the occurrence of severe complications in patients from a historical control group (*n* = 28) was 60% [37].

In our study enteral nutrition was increased very cautiously. There were no severe technical complications, solely minor complications such as occlusion or dislocation. The most common NCJ-associated complications are caused by the enteral supply of nutrients. In addition, 30% of patients undergoing upper gastrointestinal surgery develop diarrhea when fed in the early postoperative period [35]. Additionally, symptoms as nausea, vomiting, and slow gut motility can occur and can be managed by adopting the infusion rate or controlling the composition of the nutrition [35]. Just in 2% of cases there was a need for placement of a new tube through the old channel in the abdominal wall. This could be easily done via guidewire and x-ray control. Our experience goes along with the international experience [10,11,35].

In our study population the NCJ was placed for a mean time of 118 days (15 to 1150 days), which was a longer period of time compared to previous studies (12 to 35 days) [11,17,32]. After discharge, the daily amount of calories for the supplementing enteral feeding accounted 500 kcal/day in 89.2% of patients and 750 to 1.500 kcal/day in 9.8% of patients, respectively. All patients were discharged with NCJ and used it for enteral nutrition at home. In the study from Ryan et al. supplementing enteral nutrition at home was only described in 8% of patients, although oral nutrition seemed not to be adequate at the time of discharge in 40% of patients [17].

Despite long-term supplementing enteral nutrition, our patients lost weight during the first six months after surgery. Compared to the preoperative weight, our patients had a median weight loss of 7.7%. More than 10% of weight loss was detected in 39.8% of patients, a loss of more than 15% of the initial weight was observed in 13.6% of patients. Similar results were found by Heneghan et al. where 31.1% of patients undergoing esophagectomy and gastrectomy lost more than 10% of weight and 20% of patients lost more than 15% of weight in the first six months after surgery [7]. Initial overweight was an independent prognostic factor for a one year weight loss of >15% of the pre-treatment weight in 118 survivors after esophageal cancer surgery [7]. Outtara et al. observed > 15% weight loss in 25% of the patients after one year [22]. After the third postoperative year, the group of Martin et al. found a weight loss of more than 15% in 35% of patients who underwent esophagectomy [21]. Those patients did not receive supplementing enteral nutrition. These results point out the high metabolic risk in gastrointestinal cancer patients in the perioperative period. Early postoperative weight decreased up to three months, while stabilization occurred between 4–6 months after surgery, a further decline could be prevented by continuing enteral feeding supplementation. While most of the patients received 500 kcal daily, it may be speculated about the benefits of supplementing higher calorie amounts.

While computer tomography-derived body composition provides valuable information about skeletal muscle loss independent of body mass index [27], implementation in clinical practice is still pending. Bioelectrical impedance analysis (BIA) has been a proven value for the intraindividual “bedside” follow up of body composition performed under standard conditions [18]. Phase angle determined by the phase shift is a measure for cell integrity and may reflect body cell mass. Therefore, phase angle is a well-accepted parameter independent from equations validated in specific populations [38,39]. In our series the phase angle decreased after surgery but remained stable at six months after surgery. Therefore, it may be assumed that further deterioration of body cell mass could be prevented.

The study is limited by the retrospective analysis and a potential selection bias. Since the evidence for early oral food intake after esophagectomy and gastrectomy came up more recently [2,40] the restrictions within the first days were very “traditional”. Nevertheless, all calorie intake will be inappropriate for a longer period of time. Randomized trials for this group of patients focusing on the time after discharge and comparing home enteral nutrition versus regular diet and nutritional counselling are sparse. There are significant benefits regarding the incidence of malnutrition without impairment of quality of life. More supported patients may be able to complete chemotherapy [41,42]. Taking into account all these results, further randomized studies seem to be ethically acceptable only for a comparison between enteral supplementation and oral nutritional supplements (ONS).

Focusing on enhanced recovery after surgery the recent guidelines for perioperative care in esophagectomy consider enteral feeding via jejunostomy or nasojejunal/nasoduodenal tubes [43]. Our study clearly confirms the high risk for postsurgical deterioration of the nutritional status in patients undergoing upper gastrointestinal and pancreato-biliary surgery for cancer. From a metabolic and nutritional point of view the bariatric effect with considerable loss of weight and body cell mass has to be anticipated and communicated to the patient. NCJ placement is without significant harm but needs the compliance of the well-informed and cooperative patient in home care.

Furthermore, individualized home nutrition therapy requires a network in the cooperation of surgeon, general practitioner, dietitian, and home care provider. Regular monitoring of body composition and quantitative registration of oral nutrition intake is mandatory. In the future, patient reporting and dietary counselling may be improved by virtual coaching and assisted digitalized communication by chat bot [44]. Before removing NCJ, there may be a withdrawal trial for several weeks with the supplementation of ONS. In the case of weight loss during this period, enteral feeding can be resumed quickly.

## 5. Conclusions

In metabolic risk patients undergoing major surgery for cancer the option of NCJ placement offers safe access for the continuation of enteral feeding after discharge. This may be helpful for individual shared decision-making in order to attenuate postoperative weight loss and to maintain body cell mass.

## Figures and Tables

**Table 1:** Patient characteristics and clinical outcome parameters.

		Patients (*n* = 102)
Age	years	70 (39–88)
Sex	m/f	71/31
Location of the suspected carcinoma		
Stomach	% (*n*)	37.3 (38)
Esophagus	% (*n*)	27.4 (28)
Pancreas	% (*n*)	29.4 (30)
Distal bile duct	% (*n*)	3.9 (4)
Papillary	% (*n*)	2.0 (2)
Surgical procedures		
Right thoraco-abdominal esophagectomy (Ivor-Lewis)	*n*	24
Distal esophagectomy	*n*	7
Total gastrectomy	*n*	32
Distal gastrectomy	*n*	2
Gastrectomy with distal esophagectomy	*n*	1
Pylorus-preserving partial duodenopancreatectomy	*n*	14
Partial duodenopancreatectomy		22
Length of hospital stay	days	22 (11–72)
NCJ in patient	days	118 (15–1150)
Complication in Clavien-Dindo:		
No complication	% (*n*)	55.9 (57)
Grade I	% (*n*)	13.7 (14)
Grade II	% (*n*)	6.9 (7)
Grade IIIa	% (*n*)	12.8 (13)
Grade IIIb	% (*n*)	3.9 (4)
Grade IVa	% (*n*)	5.9 (6)
Grade IVb	% (*n*)	1.0 (1)
Major complication ≥ IIIb	% (*n*)	10.8 (11)
NCJ associated complications:		
no complication	% (*n*)	86.3 (88)
dislocation	% (*n*)	9.8 (10)
occlusion	% (*n*)	3.9 (4)
new placement in Seldinger technique	% (*n*)	2.0 (2)

**Table 2:** Cancer stage.

Patients *n* = 94						
Cancer Stage	Esophageal Cancer	Gastric Cancer	Pancreatic Cancer	Papillary Cancer	Distal Bile Duct Cancer	Total
UICC						
0	1	1	0	0	0	2
I	12	14	4	2	0	32
II	5	15	17	0	3	40
III	9	7	1	0	0	17
IV	1	1	0	0	1	3

**Table 3:** Complications with nutritional impact (multiple response possible).

Complication	Clavien-Dindo Classification	*n* (%)
Nausea	I	2 (2.1)
Vomiting	I	2 (2.1)
Diarrhea	I	1 (1.0)
Pancreatitis	II	2 (2.1)
Prolonged ileus	II	7 (6.9)
Pancreatic fistula	III	1 (1.0)
Anastomotic leakage	III	6 (5.9)
Anastomotic stenosis	III	4 (4.1)
Anastomotic ulcera	III	1 (1.0)
Gastrointestinal bleeding	III	2 (2.1)
Colonperforation	III	1 (1.0)
Septic shock	IV	6 (6.2)
Single or multiorgan dysfunction	IV	4 (4.1)

**Table 4:** The course of the nutritional parameters in the six months perioperatively.

	Preoperatively (*n* = 102)	1–3 Months Postoperatively (*n* = 90)	4–6 Months Postoperatively (*n* = 88)
Median BMI (kg/m^2^)	26.4 (19.2–40.0)	24.1 (18.8–39.5)	24.2 (16.3–38.9)
Underweight (<18.5 kg/m^2^)	0%	0%	2.0% (2)
Normal weight (18.5–24.9 kg/m^2^)	35.3% (36)	60.0% (54)	59.1% (52)
Overweight (25.0–29.9 kg/m^2^)	47.1% (48)	32.2% (29)	29.5% (26)
Obesity (≥30 kg/m^2^)	17.6% (18)	7.8% (7)	8.0% (8)
Median weight loss since surgery		7.7% (0–29%)	7.7% (0–24.5%)
>10% weight loss		27.8% (25)	39.8% (35)
>15% weight loss		5.6% (5)	13.6% (12)
Median phase angle	4.7° (2.7–10.3°)	4.1° (2.4–7.0°)	4.2° (1.9–6.2°)
